# Technical and Clinical Outcome of Low-Milliampere CT Fluoroscopy-Guided Percutaneous Drainage Placement in Abdominal Fluid Collections after Liver Transplantation: A 16-Year Retrospective Analysis of 50 Consecutive Patients

**DOI:** 10.3390/diagnostics14040353

**Published:** 2024-02-06

**Authors:** Robert Stahl, Max Seidensticker, Helmut Arbogast, David Kuppinger, Veronika Greif, Alexander Crispin, Melvin D’Anastasi, Vera Pedersen, Robert Forbrig, Thomas Liebig, Tim Rutetzki, Christoph G. Trumm

**Affiliations:** 1 Institute for Diagnostic and Interventional Neuroradiology, LMU University Hospital, LMU Munich, Marchioninistr. 15, 81377 Munich, Germany; robert.forbrig@med.uni-muenchen.de (R.F.); thomas.liebig@med.uni-muenchen.de (T.L.); tim.rutetzki@med.uni-muenchen.de (T.R.); christoph.trumm@med.uni-muenchen.de (C.G.T.); 2 Department of Radiology, LMU University Hospital, LMU Munich, Marchioninistr. 15, 81377 Munich, Germany; max.seidensticker@med.uni-muenchen.de (M.S.); veronika.greif@med.uni-muenchen.de (V.G.); 3 Department of General, Visceral and Transplantation Surgery, LMU University Hospital, LMU Munich, Marchioninistr. 15, 81377 Munich, Germany; helmut.arbogast@med.uni-muenchen.de (H.A.); david.kuppinger@med.uni-muenchen.de (D.K.); 4 IBE—Institute for Medical Information Processing, Biometry and Epidemiology, LMU University Hospital, LMU Munich, Marchioninistr. 15, 81377 Munich, Germany; cri@ibe.med.uni-muenchen.de; 5 Medical Imaging Department, Mater Dei Hospital, University of Malta, MSD 2090 Msida, Malta; melvin.a.danastasi@gov.mt; 6 Department of Orthopaedics and Trauma Surgery, Musculoskeletal University Center Munich (MUM), LMU University Hospital, LMU Munich, Marchioninistr. 15, 81377 Munich, Germany; vera.pedersen@med.uni-muenchen.de

**Keywords:** technical outcome, clinical outcome, CT-guided drainage, liver transplantation, fluid collection

## Abstract

Purpose: Evaluation of the effectiveness of CT-guided drainage (CTD) placement in managing symptomatic postoperative fluid collections in liver transplant patients. The assessment included technical success, clinical outcomes, and the occurrence of complications during the peri-interventional period. Methods: Analysis spanned the years 2005 to 2020 and involved 91 drain placement sessions in 50 patients using percutaneous transabdominal or transhepatic access. Criteria for technical success (TS) included (a) achieving adequate drainage of the fluid collection and (b) the absence of peri-interventional complications necessitating minor or prolonged hospitalization. Clinical success (CS) was characterized by (a) a reduction or normalization of inflammatory blood parameters within 30 days after CTD placement and (b) the absence of a need for surgical revision within 60 days after the intervention. Inflammatory markers in terms of C-reactive protein (CRP), leukocyte count and interleukin-6, were evaluated. The dose length product (DLP) for various intervention steps was calculated. Results: The TS rate was 93.4%. CS rates were 64.3% for CRP, 77.8% for leukocytes, and 54.5% for interleukin-6. Median time until successful decrease was 5.0 days for CRP and 3.0 days for leukocytes and interleukin-6. Surgical revision was not necessary in 94.0% of the cases. During the second half of the observation period, there was a trend (*p* = 0.328) towards a lower DLP for the entire intervention procedure (median: years 2013 to 2020: 623.0 mGy·cm vs. years 2005 to 2012: 811.5 mGy·cm). DLP for the CT fluoroscopy component was significantly (*p* = 0.001) lower in the later period (median: years 2013 to 2020: 31.0 mGy·cm vs. years 2005 to 2012: 80.5 mGy·cm). Conclusions: The TS rate of CT-guided drainage (CTD) placement was notably high. The CS rate ranged from fair to good. The reduction in radiation exposure over time can be attributed to advancements in CT technology and the growing expertise of interventional radiologists.

## 1. Introduction

Liver transplantation is the final therapeutic option for a considerable number of patients suffering from non-malignant and malignant liver diseases [1]. A common complication after surgery is the occurrence of intra-abdominal fluid collections [2]. These primarily consist of hematomas, seromas, bilomas, or lymphoceles [3]. They can become superinfected and can cause substantial morbidity and mortality [4]. The risk of infection is also increased in these patients due to the necessary lifelong immunosuppression.

Computed tomography (CT) serves as a suitable technique for examining fluid accumulations and determining the necessity for further medical intervention [5]. The predominant therapeutic approach involves antibiotic treatment in conjunction with percutaneous CT-guided drainage placement (CTD). The latter enables precise localization of the fluid collection and ensures the acquisition of an adequate fluid specimen for microbiological analysis and targeted antibiotic therapy [6,7,8].

CTD placement is a minimally invasive method that is widely used. It is less invasive than surgical intervention and causes a comparatively minimal burden to the patient [4,9,10]. Serious complications occur only rarely [11,12,13]. These are predominantly associated with bleeding or sepsis. The technical success rate of CT-guided drainage is thus high [14,15,16,17]. Various studies have demonstrated the clinical outcomes of CT-guided drainage placement. Most authors emphasize high clinical success rates for intra-abdominal abscesses of various origins [6,13]. However, to our knowledge, there are no studies specifically assessing fluid collections occurring in patients after liver transplantation [18].

Within this context, our study aimed to assess the long-term experience of CT-guided drainage (CTD) in patients with intra-abdominal fluid collections following liver transplantation. We were specifically interested in the following aspects: (i) the technical success rate, (ii) the occurrence of complications during the intervention, and (iii) the clinical success rate of the procedure during the post-interventional course.

## 2. Materials and Methods

### 2.1. Study Subjects

**Figure 1:**
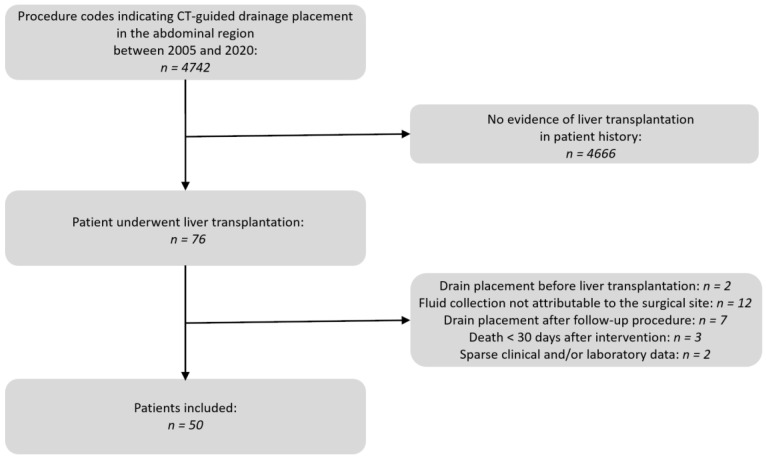
Flow chart of the patient selection process.

A comprehensive search in the Radiology Information System (RIS) of our department was performed. The focus was on the procedure code “CT-guided drainage of an intra-abdominal fluid collection” spanning the years 2005 to 2020. The results were checked for the occurrence of liver transplantation in the patient’s medical history. Medical records and OPS codes were utilized for this verification. Patients who underwent additional abdominal surgery between liver transplantation and the drain placement were excluded from this study. Additionally, fluid collections unrelated to transplantation surgery such as ascites, intramuscular abscesses of the abdominal wall, and deep pelvic collections, were not included in the evaluation. The selection process is illustrated in Figure 1.

This retrospective study received approval from the ethics committee of Ludwig-Maximilians University of Munich (number 21-0114, 2 February 2021). All interventional procedures were conducted in accordance with the principles of the Helsinki Declaration of 1964. They complied with the ethical standards of the institutional and/or national research committee, as well as its subsequent amendments or equivalent ethical standards. Informed consent was typically obtained 24 h before the intervention. In emergency cases, it was obtained immediately before the procedure, either from the patients or their legal guardians.

### 2.2. CT Imaging Protocol

The decision to perform a CTD placement was taken collectively during a multidisciplinary meeting involving radiologists and abdominal surgeons, based on contrast-enhanced cross-sectional images not older than 48 h. The following CT scanners (all manufactured by Siemens Healthineers, Erlangen, Germany) were available for the intervention: Somatom Sensation 16 (16 slice); Somatom Definition AS+ (128-slice); Somatom Definition Edge (128 slice). Each 128 slice-scanner was equipped with fluoroscopy (CARE Vision CT^®^, Siemens Healthineers, Erlangen, Germany). The patients were positioned on their back or on their side. Initially, an unenhanced CT scan with 5 mm slice thickness was performed. The purpose was to identify potential contraindications for the intervention and planning of the access path. The images were correlated with the scan on which the indication for the procedure was determined. Radiation protection measures for the interventional radiologist included the activation of Angular beam modulation (Hand Care^®^) as well as the use of thyroid shields, aprons, and eyeglasses with 0.5 mm lead equivalence. In addition, prior to applying sterile draping a shield was placed on the lower portion of the patient to reduce scattered radiation.

Pulse oximetric monitoring was performed in patients with cardiorespiratory comorbidities. Following sterile draping and skin disinfection over the designated drain entry point, local anesthesia (10 to 20 mL of 2% Mepivacaine hydrochloride) was administered. Through a minimal skin incision, the drain (Flexima^TM^ All-Purpose Drainage, Boston Scientific Corporation, Marlborough, MA, USA or Re-Solve^®^ Non-Locking Drainage Catheter, Merit Medical, South Jordan, UT, USA) was inserted and advanced to the fluid collection using the curved Trocar technique under intermittent quick-check CT fluoroscopy [16,19]. Following drain placement within the fluid collection, an unenhanced CT scan covering a minimum of 10 cm above and below the entry point along the *z*-axis was conducted to verify the accurate final drain position and eliminate immediate complications. The drain was then secured at the skin level using a suture and covered with a sterile bandage. All patients underwent clinical monitoring for a minimum of 24 h.

### 2.3. Analysis of the Pre- and Peri-Interventional Periods

Two senior IRs (R.S. and C.G.T) conducted a retrospective evaluation of patients’ imaging studies within the local PACS, radiology reports, and other medical records. Aim of this analysis was to evaluate the technical and clinical outcomes as well as complications associated with the CTD procedure over a post-interventional period of 30 days. The assessment covered a range of variables, including indications for liver transplantation, surgical techniques, immunosuppressive therapy during the intervention, predominant locations of fluid collection, interventional techniques (Trocar vs. Seldinger technique), number of drains, diameter of drainage catheters, access trajectory for drainage, and peri-interventional complications based on the SIR criteria [20]. The maximum diameter of the fluid collection and its entity was determined.

Technical success was achieved if (a) there was sufficient drainage of the fluid collection after aspiration, and (b) no peri-interventional complications occurred that necessitated surgical treatment with either a short-term (<48 h) or prolonged (>48 h) hospital stay.

Inflammatory blood parameters, including C-reactive protein (CRP), leukocytes, and interleukin-6, were measured before the intervention to identify potential superinfections. Patient radiation dose assessment was conducted separately for each stage of the intervention, namely, the pre-interventional planning CT, the sum of all intra-interventional CT fluoroscopic acquisitions, and the post-interventional control CT. These data were provided by the CT scanner in the form of a dose report. According to Kloeckner et al. [21], the dose-length product (DLP [mGy·cm]) was used for this analysis.

### 2.4. Analysis of the Post-Interventional Period

To demonstrate the effect of CTD placement, we initially identified the subgroup of patients who had no additional surgical interventions or complications in the Hospital Information System (HIS). The temporal evolution of inflammatory parameters, including CRP, leukocyte count, and interleukin-6, was then analyzed over a 30-day period following the intervention in this subgroup. Success was defined if these values were initially elevated and either normalized or reduced by at least 50% from their baseline. In addition, we investigated whether parameters of liver function and liver damage also changed after the CTD placement. Therefore, the 30-day course of the blood levels of albumin, total serum bilirubin, cholinesterase and the International Normalized Ratio (INR) were analyzed to assess liver function. Levels of gamma glutamyltransferase (GGT), aspartate aminotransferase (AST), alanine-aminotransferase (ALT) and antithrombin were used as markers for liver damage.

Furthermore, clinical success was defined when no surgical revision associated with the intervention was required. Microbiological analyses were conducted on the fluid discharged through the drainage catheters.

In addition, the variables collected in this study were divided into two subgroups of the patient population where the placement was technically successful and no reoperation occurred. In one group, a decrease in initially elevated inflammatory parameters was observed within 30 days, while in the other this was not the case. Variables showing significant differences were identified as potential prognostic parameters for a clinically successful drainage placement.

The number of days the drainage remained in the patient was recorded.

### 2.5. Statistical Analysis

First, the distribution of discrete and continuous data was checked for normality. Shapiro–Wilk tests and histograms were used for this purpose. Normally distributed variables are then shown as the mean ± standard deviation (SD). Variables that deviate from the normal distribution are presented with their median values (25th to 75th percentile) and their range.

In the case of binary variables (e.g., proof of germs in the fluid collection) and categorical variables (e.g., visual appearance of the fluid collection) contingency tables were calculated. The independence of these variables was assessed with Chi2 or Fisher’s exact tests. Fisher’s exact test was used for fourfold tables, otherwise Chi2 tests were conducted. Post hoc Fisher’s exact test with Bonferroni correction was performed when statistically significant results in the Chi2 tests occurred.

To assess differences in radiation exposure between the two distinct time periods, Mann–Whitney tests for independent samples were used.

Generalized linear mixed models (GLMM) were utilized to investigate the temporal changes in inflammatory parameters during the initial 30 days following the intervention. The fixed effect parameter was the number of days after the intervention. The random intercept was the subject ID repeated by days. The values were log-transformed prior to analysis to achieve normal distribution.

The analysis was conducted using R (R Core Team (2022). R: A language and environment for statistical computing. R Foundation for Statistical Computing, Vienna, Austria. URL https://www.R-project.org/, version 4.2.3, accessed on 2 September 2023). A *p* value of <0.05 was considered statistically significant (significance level of α = 0.05).

## 3. Results

We included 50 patients (13 females) who had undergone CT-guided drainage following liver transplantation between 2005 and 2020. Details regarding the patient cohort are outlined in Table 1.

The most common indication for liver transplantation was alcohol-induced cirrhosis (n = 20, 40.0%), which was accompanied by additional hepatitis C virus (HCV) infection in two cases (4.0%). In 12 (24.0%) patients with cirrhosis additional hepatocellular carcinoma (HCC) was present. The second most frequent disease group was autoimmune hepatitis (n = 6, 12.0%) followed by acute liver failure (ALF) (n = 3, 6.0%). Cholestatic changes in the biliary tract and Budd–Chiari syndrome were the cause in two cases each (4.0%).

The majority (n = 41, 82.0%) were “whole-liver” transplants, meaning that the whole organ was used. In nine (18.0%) of the operations, only one half of the liver was transplanted (“split-liver”).

Most often, the operation was performed using the piggyback technique (n = 32, 64.0%) or modified according to Belghiti (n = 9, 18.0%). Resection of the recipient vena cava was performed in nine cases (18.0%).

In six patients (12.0%), it was the second transplantation after a previous graft failure.

In six cases (12.0%), the donor liver had an accessory or aberrant hepatic artery that required anastomosis during the backtable preparation before implantation.

The most frequent type of biliary anastomosis was end to end (n = 37, 74.0%). Less common was an end-to-side anastomosis (n = 4, 8.0%). A bilodigestive anastomosis was constructed in nine patients (18.0%).

In five patients (10.0%), additional abdominal surgical procedures were performed during the transplantation (allogeneic kidney transplantation in two patients (4.0%); and intestinal resections in three patients (6.0%)).

### 3.1. Pre- and Peri-Interventional Analysis

Overall, 91 intervention sessions were performed in our cohort of 50 patients (mean ± SD per patient: 1.8 ± 1.3). The distribution of the time points was very heterogeneous: most procedures were conducted within the first year after surgery (n = 67, 73.6%). In the second year, there were n = 7 (7.7%) CTD placements. In the third, fourth and sixth years after transplantation, there were n = 2 (2.2%) interventions each. Furthermore, very late interventions were documented in the 9th (n = 5, 5.5%) and 23rd (n = 6, 6.6%) year after transplantation.

At the time of the intervention, tacrolimus was the most frequently used immunosuppressant (n = 63, 70.0%). In 8 of these cases, mycophenolate was also administered. Cyclosporin A was given in 22 cases (24.4%), in combination with mycophenolate in 7 cases and combined with sirolimus in 4 cases. Sirolimus as sole therapy was present in five (5.6%) interventions. In 44 patients who received tacrolimus, concomitant therapy with cortisone was given. A total of 12 patients with cyclosporin A therapy also received concomitant cortisone therapy.

A total of 116 fluid collections were treated with a total of 124 drain placements. A total of 67 (57.8%) lesions were localized in the liver, of which 64 (55.2%) were located in the parenchyma and 3 (2.6%) were subcapsular. A total of 47 (40.5%) collections occurred in compartments adjacent to the liver. In two (1.7%) cases, an external PTCD was placed in centrally congested bile ducts.

In 85 interventions (96.6%), the Trocar technique was utilized while the Seldinger technique was employed in three procedures (3.4%). Data on the technique used were not available for three interventions. On average, 1.4 drainages (SD: ±0.6) were inserted per intervention. Drainage diameters varied with 7.5 French (F) in 8 cases (7.1%), 8F in 38 cases (33.9%), 10F in 54 cases (48.2%), 12F in 11 cases (9.8%), and 14F in one case (0.9%). Information on the diameter was not available for 12 drainages. A direct transabdominal access path for drainage catheter placement was chosen in 82 drains (66.1%), while a transhepatic access path was selected in 42 drainage placements (33.9%). Further details on drainages and intervention techniques are provided in Table 2.

A total of 83/116 (71.6%) collections occurred in the first year after transplantation. This comprised the majority of abscesses (n = 27/44, 61.4%), bilomas (21/37, 56.8%), seromas (n = 6/8, 75.0%) and hematomas (n = 10/11, 90.9%). From the ninth year onwards, 5/44 (11.4%) abscesses and 10/37 (27.2%) bilomas were found. There were no more hematomas and seromas.

**Figure 2:**
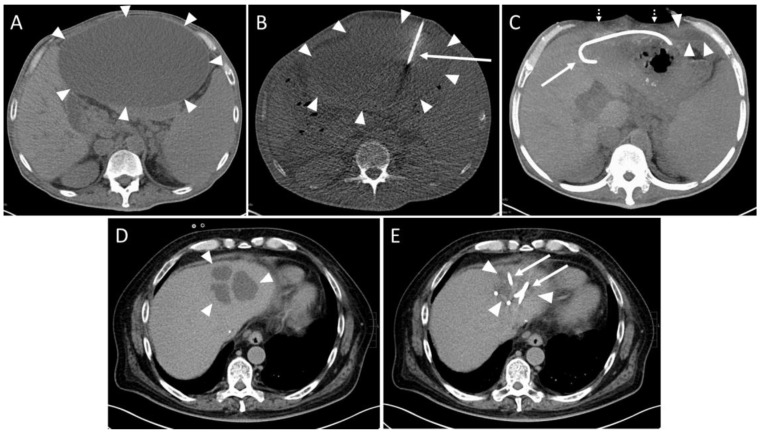
Example of a routine CT-guided drainage placement without complications. A 50-year-old man with history of liver transplantation due to alcohol-induced cirrhosis. Five weeks after surgery, an increase in transaminases and serum bilirubin was observed. Later, a palpable mass and upper abdominal pain developed. (**A**) CT planning scan 59 days after transplantation showed a large subcapsular fluid collection with mass effect encompassing the anterior liver margin (arrowheads). (**B**) CT fluoroscopy scan: An 8F drain (arrow) placement in the collection (arrowheads) via a left anterior transabdominal access using the Trocar technique was performed. (**C**) Maximum-intensity projection (25 mm slab thickness) of the CT control scan revealed a marked reduction in the mass effect. It was possible to aspirate 3000 mL of yellow-green fluid, resulting in almost complete drainage of the formation. Only a small residue (arrowheads) remained in the left recessus. Patient reported instant relief of symptoms (dotted arrows: collapsed abdominal wall, arrow: drain). No signs of complications such as organ penetration or bleeds are visible. Microbiological analysis revealed a sterile biloma. (**D**) Eight years later, the patient was readmitted due to cholangitis of the left liver lobe with several abscesses (arrowheads). (**E**) Again, CT-guided placement of two drains (arrows) could be successfully performed. The additional administration of pathogen specific antibiosis led to a decrease in inflammatory parameters. Arrowheads: Abscesses.

A total of 64/116 (55.2%) collections were intrahepatic. An extrahepatic localization was found for 52 collections (44.8%). Analyzing the drainage fluid, 44/100 collections (44.0%) were abscess formations, 37 (37.0%) were biloma, 11/100 were hematomas and 8/100 were seromas. Intrahepatic abscesses were more prevalent, accounting for 65.9% (n = 29/44), compared to extrahepatic locations, which constituted 34.1% (n = 15/44). However, this difference did not achieve statistical significance (*p* > 0.05). Hematomas were significantly (*p* < 0.01) more frequently extrahepatic (n = 10/11, 90.9%) than intrahepatic (n = 1/11, 9.1%). In 85/91 (93.4%) interventions, the placement of the drainage was technically successful (Figure 2).

**Figure 3:**
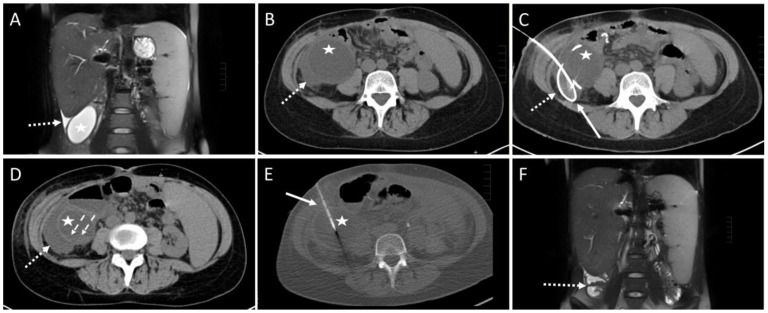
Example of a CT-guided drainage placement involving minor complications in accordance with SIR (Society of Interventional Radiology) guidelines. A 30-year-old female with a history of liver transplantation due to primary sclerosing cholangitis. Postoperative ultrasound follow-up revealed a cystic subhepatic fluid collection that recurred over months and was considered to be a biloma. The patient was symptom-free. However, approximately 10 months after transplantation the patient complained of increasing feeling of pressure and pain in the right lower quadrant of the abdomen. (**A**) MRI scan (coronary reconstruction) revealed a roundish thin-walled fluid collection (asterisk) in the subhepatic paracolic gutter, which, in the context of the described symptoms, was an indication for elective CTD. Additionally, note the small amount of ascites in the perihepatic and perisplenic gutter (dotted arrow). (**B**) Pre-interventional CT planning scan shows the cystic fluid collection (asterisk) and the surrounding ascites (dotted arrow). (**C**) Post-interventional control scan: Due to a highly rigid wall an 8F drain (solid arrow) could only partially be inserted via the Seldinger technique. The proximal end is dislocated in the right subhepatic gutter (dotted arrow). (**D**) Post-interventional unenhanced CT control scan after partial aspiration and removal of the drain exhibits a small hemorrhage (dashed arrows, density: 22 Hounsfield units) at the bottom of the cavity of the cystic formation (asterisk). Further, a slight increase in the extent of the fluid collection (dotted arrow, compare to figure (**B**)) in the gutter was observed, most probably due to fluid loss through the drain access hole into the gutter. Microbiological analysis was negative. (**E**) CT fluoroscopy: After 4 weeks, a new approach using the Trocar technique was attempted and a successful drainage placement was obtained. Asterisk: cystic fluid collection. Arrow: 8F drain. However, 6 months later, due to recurrence of the fluid-filled collection with vena cava compression, the formation was operatively resected. Histopathological examination revealed a cholangiocele of the bile duct system. (**F**) Postoperative MRI scan 4 month after surgery shows only parts of colon and small amounts of ascites subhepatically (dotted arrow) at the original site of the cholangiocele.

One intervention had to be aborted since the patient complained of pain exacerbation. No fluid could be aspirated in one session. In one case, the aspirate contained fresh blood. The drain was removed and subsequent control imaging examinations showed no active bleeding (SIR Grade A). In three cases, a small pneumothorax was observed in the control scan (SIR Grade A). The punction of a cystic fluid collection yielded a small hemorrhage without the need for therapy in one patient (SIR Grade A, Figure 3).

**Figure 4:**
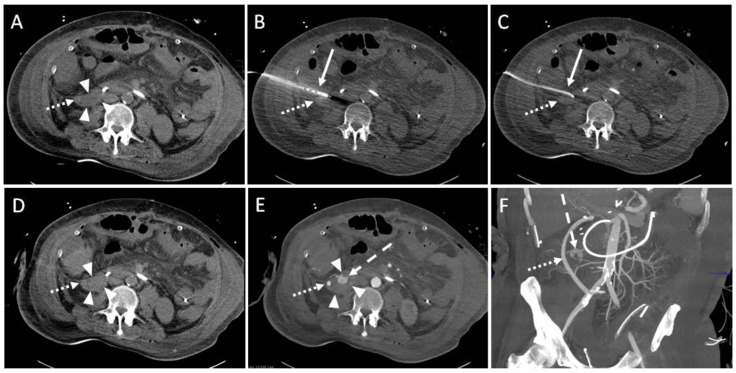
Example of a CT-guided drainage placement with significant complications following SIR guidelines. A 47-year-old male with history of liver transplantation due to alcoholic cirrhosis. After six weeks, an increase in inflammatory parameters was observed. (**A**) CT planning scan 52 days after transplantation: a fluid collection (arrowheads; density: 6 Hounsfield units (HU)) encompassing the bypass (dotted arrow) between right common iliac artery and hepatic artery in the right paracolic gutter. (**B**) Using lateral access via the retrocolic space, a 10F pigtail drainage (solid arrow) is placed under CT fluoroscopy. Dotted arrow: drainage trajectory anterior to the bypass. (**C**) After removal of the Trocar, a correct position of the drain (solid arrow) was shown. Several milliliters of serous fluid could be aspirated. Dotted arrow: bypass. (**D**) An unenhanced CT control scan revealed increased density (30 HU; compare to figure (**A**)) of the fluid collection (arrowheads) in the right paracolic gutter suspicious of acute bleeding. Dotted arrow: bypass. (**E**) Enhanced follow up CT scan 10 min after drain placement shows contrast agent extravasation (dashed arrow) originating from a branch of the right colic artery. Due to the arterial contrasting, the bypass (dotted arrow) is now clearly delineated. Arrowheads: fluid collection. (**F**) Coronary maximum-intensity projection (40 mm) showed an intact, inconspicuous bypass (dotted arrow). Dashed arrow: contrast agent extravasation. The injured vessel was subject to successful immediate revision surgery.

In one intervention, large amounts of blood were aspirated through the drainage. A gelatin slurry (Gelofam) was applied via the drainage which successfully stopped the bleeding (SIR Grade C). After one intervention, an immediate revision surgery was re-quired due to an accidentally injured artery during the CTD placement (SIR Grade D, Figure 4).

In summary, five (5.5%) minor and two (2.2%) major complications were observed (Table 3). The technical success rate yielded 93.4% (85/91).

Baseline values (median [25%, 75% quartile]) were 7.9 [4.7, 14.3] mg/dL for CRP; 7.5 (4.7, 12.3) × 10^9^/L for leukocytes, and 241 [83.4, 431.5] pg/dL for interleukin-6.

Increased levels at the day of the intervention were detected in 97.5% (77/79) for CRP (>0.5 mg/dL), in 34.9% (29/83) for leukocytes (>9.8 × 10^9^/L), and in 100% for interleukin-6 (>5.9 pg/dL) among the 35 patients where this parameter was determined.

**Figure 5:**
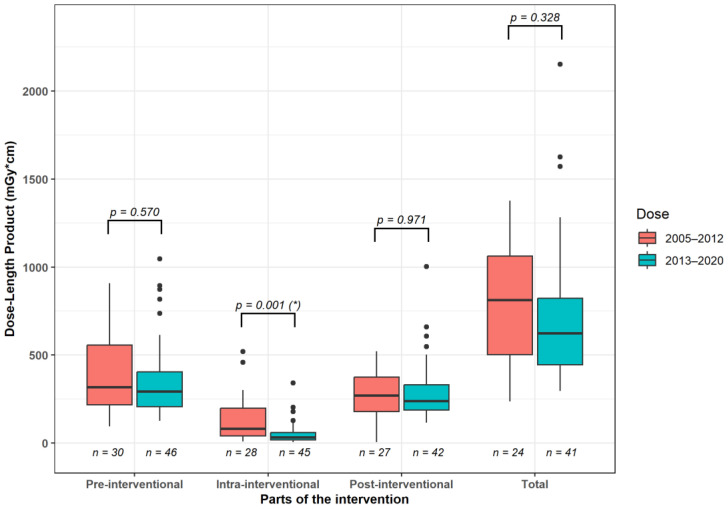
Median radiation dose differences between 2005–2012 and 2013–2020 for the individual CT scan steps and the entire procedure. *: significant intergroup differences.

The analysis of dose values across all intervention phases showed that the DLP for the fluoroscopy component was significantly lower (*p* = 0.001) in the later time period compared to the preceding years (years 2005 to 2012 median [25%, 75% quartile]: 80.5 [40.0; 197.0] mGy·cm vs. years 2013 to 2020: 31.0 [18.0; 59.0] mGy·cm; Figure 5). DLP values tended to be lower (*p* > 0.05) in the second observation period compared to the first observation period for both the pre-interventional planning scan and the post-interventional control scan (years 2005–2012: planning scan 317.0 [217.5, 556.8] mGy·cm, control scan: 270.0 [179.0, 375.0] mGy·cm vs. years 2013–2020: planning scan: 293.0 [205.8; 404.5] mGy·cm; control scan: 238.5 [187.3, 330.0] mGy·cm). Consequently, the cumulative DLP values for the entire intervention showed a trend (*p* = 0.328) to be lower in the years 2013–2020 (623.0 [445.0; 822.0] mGy·cm) compared to the years 2005–2012 (811.5 [502.3; 1061.5] mGy·cm).

### 3.2. Post-Interventional Analysis

The clinical follow-up within 30 days was as follows: After CTD placement, drain dislocation occurred in five patients. In four patients Endoscopic Retrograde Cholangi-opancreatography (ERCP) was conducted. Four patients suffered from graft failure. Nine patients developed sepsis. Within 60 days after intervention, five patients deceased due to septic multi-organ failure. In 36 patients, at least one additional CTD placement was per-formed within 60 days.

In terms of achieving a successful clinical outcome as indicated by the necessity for reoperation, surgical revision was necessary in three patients (6.0%) due to insufficient drainage of the fluid collection.

**Figure 6:**
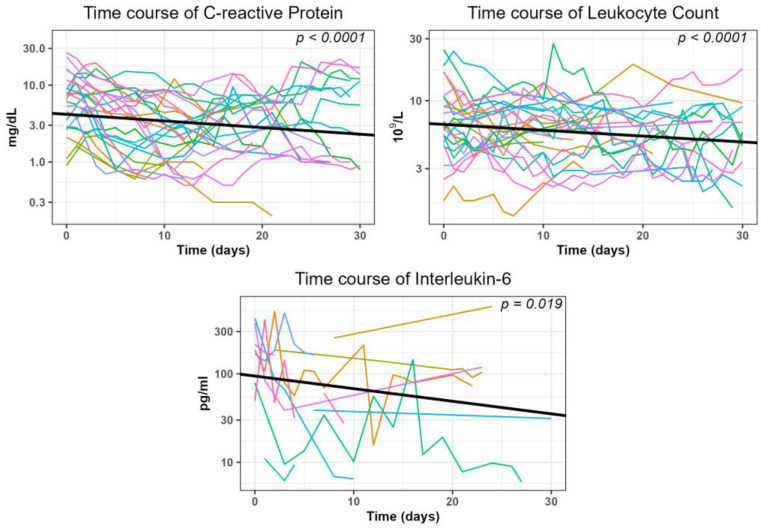
Generalized linear mixed model (GLMM) analysis illustrates the trend of inflammatory parameters within 30 days after the intervention.

In the subgroup of patients who did not undergo additional surgical or interventional therapies within 30 days after the intervention, the inflammatory parameters significantly (*p* < 0.05, Figure 6 and Table 4) decreased during this period. The average reduction in log-transformed values was −0.008621 mg/dL for CRP, −0.00450 × 10^9^/L for leukocytes, and −0.01423 mg/dL for interleukin-6, respectively.

The liver function parameters albumin and cholinesterase in this subgroup showed a significant (*p* < 0.0001) increase (albumin: 0.00272 g/dL, cholinesterase: 0.00524 kU/L; Supplementary Figure S1 and Supplementary Table S1).

ALT was the only parameter representing liver damage that showed a significant de-crease (−0.0066 U/L). The other laboratory parameters studied did not change significantly (Supplementary Tables S2 and S3).

Clinical success in the whole collective was defined by the reduction in elevated inflammatory markers either by at least 50% or by reaching the normal range. This was attained in 52 out of 77 interventions (67.5%) for CRP, with a median duration of 5.0 (25%, 75% quartile: 3.75, 8.0) days. For leukocytes, clinical success was observed in 23 out of 29 interventions (79.3%), with a median duration of 3.0 (2.0, 5.5) days. Regarding interleukin-6, clinical success was achieved in 20 out of 35 interventions (57.1%), with a median duration of 3.0 (1.0, 7.25) days.

Microbiological specimens from wound secretions were successfully obtained in 111 lesions (95.7%), and positive confirmation was observed in 70 cases (63.0%).

The most commonly identified bacterial strains were Enterococci, found in 46 lesions. Candida, the predominant pathogenic fungus, was present in 46 lesions as well. An overview of the microbiological findings is presented in Supplementary Figure S2.

A differentiation of the success rate between the fluid collections with and without positive germ detection is presented in Table 5.

In infected fluid collections, success rates tended to be higher compared to non-infected ones. The highest rate (78.9%) was observed for the parameter leukocyte count.In 66 out of 91 interventions (72.5%), there was both technical and clinical success, meaning the avoidance of re-operation and a reduction in potentially elevated inflammatory parameters within 30 days.

In 65 cases, information on the appearance of the drainage fluid was available (Supplementary Table S4). The fluids most commonly appeared macroscopically purulent (15/65, 23.1%), which was statistically significant (*p* < 0.001). Moreover, in cases where this visual appearance was present, pathogens were significantly (*p* < 0.0001) more frequently detected compared to non-purulent collections (positive in 14 out of 15 cases, 93.3% vs. negative in 1 out of 15 cases, 6.7%). A chylous-like appearance occurred only once, which was significantly the least frequent (present in 1 out of 65 cases, 1.5%).

Patients who had a successful clinical course with decreasing inflammatory parameters had a significantly higher incidence of alcoholic liver damage with or without HCC as an indication for transplantation compared to those patients where values did not decrease (*p* < 0.01; Supplementary Table S5). The analysis of the aspirate revealed a significantly higher prevalence of an abscess or biloma (*p* < 0.01; Supplementary Table S6). Additionally, a right lateral access approach was significantly more frequently chosen (*p* = 0.042; Supplementary Table S7). In patients with a clinically unsuccessful reduction in inflammatory parameters, the aspirate appeared significantly more frequently bloody (*p* = 0.002), and there was a significantly higher incidence of hematoma (*p* = 0.08).

The median indwelling time of the drainage was (median [25%, 75% quartile]) 8 (6; 19) (ranging from 0 to 47) days.

## 4. Discussion

The outcomes of CT fluoroscopy-guided drainage for fluid collections in patients following liver transplantation were assessed in our study. We performed a comprehensive analysis on a total of 91 interventions carried out in 50 patients over a 16-year period.

Abdominal fluid collections represent a frequent complication occurring in as many as 76% of cases [2,22,23]. Winston et al. [24] identified the complexity of the surgery and the insertion of the transplanted organ into a possibly infected abdomen in patients with a reduced general condition as risk factors at a very early stage. Possible risk factors in the later postoperative phase may lead to a superinfection of these lesions or the development of further infected fluid collections partly even years later [25]. According to Czerwonko et al. [12] the most important contributors are immunosuppression, malnutrition, diabetes, biliary instrumentation, liver ischemia, and a reconstructed biliary anatomy. This is also reflected in our study: Although most collections appeared within the first year after the operation these occurred even up to 23 years later. In the early phase, all entities of the fluid collections were found. However, hematomas and seromas as typical side effects of surgery were not present in our group in the late phase. Here, we observed only abscesses and bilomas.

The insertion of a CTD can be associated with a range of complications of varying degrees of severity. Its main spectrum includes pneumothorax and bleeding, but sepsis or even death can also occur [5,16,19,21].

Akinci et al. demonstrated in a larger case series of 291 patients with drainable intraperitoneal abscesses a peri-complication rate of 12% [11]. In our study, the technical success rate was slightly lower. We observed seven patients (7.7%) with two classified as major complications according to the SIR guidelines [20]. This is in agreement to Wallace et al. [8] suggesting a threshold of less than 10%. A total of 93.4% of the interventions in our study were technically successful. This value is within the range from 88.9% to 100% found by other authors [7,10,13] and is also within the range of 93.0% to 100%, which we have achieved in studies involving other patient cohorts following abdominal interventions [26,27,28,29]. The most serious complication observed in our patient cohort was severe hemorrhage in one case, requiring immediate surgical intervention. The probable cause was presumed to be patient movement occurring between the acquisition of the planning scan and fluoroscopy scans. We conclude that a reduction in the incidence of complications may involve a more rigorous patient selection process and a broader utilization of conscious sedation or general anesthesia, particularly in the early postoperative period.

Clinically successful drainage placement in our study was defined by two criteria. One of them was the avoidance of surgical revision due to insufficient drainage. Our rate yielded 94.0% which is concordant with findings from other studies applying similar criteria in patients with multiple abdominal resections: The same ratio as by us could be achieved by Asai et al. [6]. Their applied criterion for success was the patient’s healing and discharge from the hospital without an indwelling drainage. However, with 47 patients they examined about as many as we did but the total number of 54 drainage placements was only 60% of our value. In addition, their patient population comprised unselected abdominal deep tissue abscesses. Laganà et al. [13] studied 107 procedures in patients with abdominal and pelvic abscesses, although their focus was on ultrasound-guided targeting. In the 37 patients in their subgroup with CTD placement, a clinical success rate of 92% could be achieved. They defined clinical success as the progressive shrinkage of the collection by more than 50%. The above-mentioned study by Akinci et al. [11] comprised a large number of cases with 291 patients with intraperitoneal abscesses. The overall success rate was 91%. Their definition of clinical success was more intricate: they categorized CTD placement as successful (i) if healing without the need for additional interventions was achieved, (ii) if further surgeries were performed only due to the primary disease and not related to the drained lesion or when CTD placement was conducted palliatively because of an incurable primary disease. However, comparable to [13] and in contrast to our study, drainage placements with other imaging modalities such as fluoroscopy and ultrasound were also involved.

The second criterion for clinical success in our group was the decrease in laboratory values which are usually elevated in inflammation. Here, either reaching the normal value or reducing an elevated value by at least half was considered a success. This was most frequently met for leukocyte count and CRP (77.8% and 64.3% of interventions, respectively) with a somewhat lower decrease observed for interleukin-6 (54.5%). However, interleukin-6 was determined in relatively few patients (n = 15). This can be explained by the fact that the determination of this parameter is relatively expensive compared to leukocyte count and CRP.

The course of inflammatory parameters after CTD placement was previously examined by our group in cohorts after abdominal surgeries. We studied patients after pancreatic resection [27], liver resection [26], and colorectal resection [28], as well as after lymphocele resection following prostatectomy [29]. The resulting success rates for CRP ranged between 83.3% and 94.4%, yielded between 78.4% and 100% for leukocytes, and were 87.5% for IL-6 (this parameter was only analyzed after pancreatic resections). For the patients in the current study, the values are somewhat lower. We attribute it to the fact that patients after liver transplantation are often critically ill compared to those in the other cohorts. They frequently undergo intensive medical care and their acute phase parameters are more exposed to various influences. The time at which success was achieved was within one week for all parameters. Leukocyte count and interleukin-6 responded the fastest, with a median value of 3 days each. CRP took slightly longer, with a median of 5 days. The simultaneous significant increase in albumin and cholinesterase levels as well as the decrease in ALT values in this subgroup can be seen as additional confirmation of the effectiveness of the CTD placement.

The combined rate for technical and clinical success yielded 72.5%. This relatively lower value can be explained by variations in how success was defined in other studies as stated above. Specifically, the reduction in inflammatory parameters introduced by us has not been utilized by other authors.

Our subgroup analysis based on the decrease in inflammatory parameters identified prognostic factors for clinical success. Favorable factors included the presence of alcoholic liver damage with or without HCC as an indication for transplantation. A possible explanation is that, unlike severe systemic conditions such as autoimmune diseases, these are local pathologies that can be significantly remedied with transplantation. Consequently, the patients may find themselves in a relatively better condition. It was also favorable when the cause of fluid accumulation was an abscess or biloma. In contrast, an unfavorable factor was identified if hematoma was the cause, associated with a correspondingly bloody appearance of the aspirate. We conclude that in the case of abscesses/bilomas, CTD placement achieves better efficacy through the drainage of pus or the drain of bile fluid compared to the mechanical relief of a hematoma or seroma, most probably due to an increased viscosity. Furthermore, placement of the drainage through a right lateral transabdominal access route was also found to be prognostically favorable. Possibly, the drainage is better tolerated by the patient in this case, or there is improved nursing access, which could potentially result in better drainage efficiency or a slightly longer duration of the drain.

In contrast to the two-stage Seldinger technique, the Trocar technique consists of only one step. The advantages are ease of handling and time efficiency compared to the Seldinger technique [17]. It was therefore used for most of our patients. In analogy to Halliday et al. [2], the majority of collections in our study were abscesses which were localized in the liver parenchyma (55.2%). This represents the relatively high proportion of transhepatic approaches (33.8%) in our interventions.

Since the peritoneal reflections around the liver are divided during transplantation [2] extrahepatic fluid can accumulate to other sites as commonly observed in non-transplanted patients [30]. In our collective, extrahepatic lesions were most frequently localized prehepatic, subhepatic and perihepatic next to the liver hilum (29.3.%). Therefore, most of the transabdominal approaches (60.5%) were conducted through the ventral and right lateral sector of the abdomen (transverse section) [31].

The dosimetric analysis was performed by evaluating the DLP for the various intervention parts and dividing our observation period into two time spans. For both time periods, the fluoroscopy section was the one that accounted for the smallest proportion of the total intervention dose. It yielded 9.9% for the years 2005–2012 and 5.0% for the years 2013 to 2020. This result is consistent with the study by Kloeckner et al. [21]. In a study of 1284 patients on CT intervention types on various body sections, they showed that only around 15% of the dose is incurred during the actual intervention part. The majority is generated during the pre- and post-inventional scans. Furthermore, the DLP values for the fluoroscopic part of the CT intervention were statistically significantly reduced in the second period compared to the first period. However, DLP values for the pre- and post-interventional CT scan as well as for the entire intervention only tended to be lower in the second part (years 2013–2020) than in the first part (years 2005–2012). Klöckner et al. conducted their study in 2013 and recommended a threshold of 942 mGy·cm for DLP in abdominal drain placement procedures. The median values were low in the first section of the observation period of our study and significantly below this threshold in the second section (years 2005–2012: 811.5 mGy·cm, years 2013–2020: 623.0 mGy·cm). Yang et al. [32] conducted a single-center analysis, involving 1977 abdominal CT-guided interventions from 2012 to 2017. They reported a median DLP value of 1043 mGy·cm in cases where one drain was inserted. This threshold was also met in our study.

The development of CT scanner technology has incorporated various approaches that contributed to the reduced dose in the second half of our study [14,15,16]. These include (i) tube current modulation to change the tube current time product without negatively influencing the signal to noise ratio; (ii) iterative image reconstruction to reduce patients exposure time; (iii) increase in the number of detector rows in multi-slice CTs for simultaneous acquisitions of adjacent body sections and (iv) improvements in CT fluoroscopy to visualize the needle during the puncture procedure almost in real time. As a result, two 128 scanners were available for interventions in our department in the second half of our study period. Both featured angular beam modulation [33] by switching off radiation between the ten and two o’clock positions of the X-ray tube, and one of them was additionally equipped with a Stellar Detector [34]. Its principle is the integration of electronic components necessary for the detection of an X-ray beam in a specific integrated circuit. This reduces the signal path, which leads to a lower SNR and a reduction in dose. Another factor is the fact that as the use of CTD progressed more experience was gained in handling the method. This learning curve led to a faster completion of the procedures, so the Quick-check method was increasingly used [35]. Here, repeated images of individual CT fluoroscopic images are taken after each change in needle or table position. Continuous fluoroscopy is thus avoided, which results in dose savings.

In the case of infected collection, Enterococci, Staphylococci, and *E. coli* were the bacteria most frequently found. The most common fungus was Candida. These findings are in agreement with pathogens commonly associated with intra-abdominal infections [36,37]. The relatively high proportion of Candida may be an expression of an opportunistic infection due to an altered immune defense caused by the immunosuppressive therapy [38]. We conclude from this that the additional presence of a fungal infection in this patient population should be considered at an early stage if the clinical or laboratory inflammation values persist after a successful CTD placement despite germ-adapted or broad-spectrum antibiotic therapy.

In summary, CT-guided drainage demonstrated fair to good success rates in managing postoperative abdominal fluid collections following liver transplantation. In comparison to the established clinical use of US-guided drainage placement, this method offers the advantage of providing depiction of postoperative and thus often distorted abdominal site. The improved delineation of air-overlaid bowel and vessels makes it suitable for deep and small lesions, requiring less subjective experience from the interventionalist. The main drawback compared to US-guided drainage placement is that both the patient and the interventionalist are exposed to ionizing radiation. Additionally, the CT scanner is usually located in the radiology department, potentially requiring longer transport distances for critically ill patients. US-guided drainage is also the more cost-effective method than CTD.

There are several reasons why limitations of our study might exist. First, data presented in the study were gathered retrospectively from a single institution over a span of 16 years. Second, the success of CTD placement can be defined in various ways. This becomes evident by analyzing studies in the literature where various approaches exist to describe the effectiveness of CTDs. Therefore, we have opted for a combination of clinical and laboratory parameters that reflect the realities of clinical practice. However, particularly in critically ill patients with the potential need for intensive medical care the laboratory parameters are subject to various influences. Their alterations may not necessarily reflect the changed situation following the drainage of an intra-abdominal fluid accumulation. Third, a significant portion of patients had to be excluded from the retrospective analysis due to the absence or incompleteness of data.

## 5. Conclusions

The insertion of CT-guided drainage in patients with symptomatic fluid collections in the abdomen post-liver transplantation yields a highly successful technical outcome. A noteworthy clinical response, marked by a decrease in inflammatory parameters and a reduced necessity for reoperations, is also evident. Severe complications are exceptionally rare. Advances in CT scanner technology in recent years have resulted in a substantial decrease in radiation exposure, especially in the CT fluoroscopy component, and a notable trend towards a reduced total radiation dose for the entire procedure.

## Figures and Tables

**Table 1:** Details of 50 patients who underwent percutaneous drainage of fluid collections following liver transplantation under CT fluoroscopy guidance.

Variable	Numbers (%)
Sex	
Female	13 (26.0)
Male	37 (74.0)
Indications for liver transplantation	
*Cirrhosis (reasons; complications)*	*36 (72.0)*
Ethyltoxic	14 (28.0)
Ethyltoxic; HCC	4 (8.0)
Ethyltoxic + HCV	1 (2.0)
Ethyltoxic + HCV; HCC	1 (2.0)
HBV + HDV	2 (4.0)
HBV + HDV; HCC	1 (2.0)
HBV	1 (2.0)
HBV; HCC	1 (2.0)
HCV	2 (4.0)
HCV; HCC	5 (10.0)
Drug induced	1 (2.0)
Alpha-1 Antitrypsin Deficiency	1 (2.0)
Cryptogenic	2 (4.0)
*Tumor only*	*1 (2.0)*
HCC	1 (2.0)
*Fulminant hepatic failure*	*3 (6.0)*
Drug induced	1 (2.0)
Unknown reason	2 (4.0)
*Autoimmune*	*6 (12.0)*
Autoimmune hepatitis (AIH)	2 (4.0)
Primary sclerosing cholangitis (PSC)	1 (2.0)
AIH/PSC overlap	1 (2.0)
Primary billiary cirrhosis	2 (4.0)
*Cholestatic*	*2 (4.0)*
Secondary sclerosing cholangitis	1 (2.0)
Secondary billiary cirrhosis	1 (2.0)
*Other reasons*	*2 (4.0)*
Budd–Chiari syndrome	2 (4.0)

AIH: autoimmune hepatitis; HBV: hepatitis B virus; HCC: hepatocellular carcinoma; HCV: hepatitis C virus; HDV: hepatitis D virus; PSC: primary sclerosing cholangitis.

**Table 2:** Details on the intervention site, immunosuppressive treatment, drains and techniques used during the 91 CTD intervention sessions.

Time from surgery to first intervention (days):	46 (24, 128) (7–8212) ^1^
Age at intervention (years)	59.9 ± 10.8 (27–68) ^2^
Immunosuppressive treatment	Count ^3^
Tacrolimus	55 (61.1)
Tacrolimus + mycophenolate	8 (8.9)
Cyclosporin A	11 (12.2)
Cyclosporin A+ mycophenolate	7 (7.8)
Cyclosporin A+ sirolimus	4 (4.4)
Sirolimus	5 (5.6)
Max. diameter of the fluid collection (cm)	7.1 (4.4, 11.0) (2.0–20.2) ^1^
CT signs of infection	53 (58.3%) ^3^
Predominant location of the fluid collection	Count ^3^
Intrahepatic right liver lobe	43 (37.1)
Intrahepatic left liver lobe	13 (11.2)
Intrahepatic central	8 (6.9)
Subcapsular right liver lobe	3 (2.6)
Centrally congested bile ducts	2 (1.7)
Prehepatic	8 (6.9)
Subhepatic	16 (13.8)
Perihepatic medial	10 (8.6)
Perihepatic lateral	5 (4.3)
Retrohepatic right	2 (1.7)
Retrohepatic left	2 (1.7)
Gallbladder bed	2 (1.7)
Subphrenic	2 (1.7)
Drainages per intervention	Count ^3^
1	63 (69.2)
2	24 (26.4)
3	3 (3.3)
4	1 (1.1)
Diameter (French)	Count ^3^
7.5	8 (7.1)
8	38 (33.9)
10	54 (48.2)
12	11 (9.8)
14	1 (0.9)
Technique	Count ^3^
Trocar	85 (96.6%)
Seldinger	3 (3.4%)
Access path	Count ^3^
Transabdominal ventral	29 (23.4)
Transabdominal right ventrolateral	13 (10.5)
Transabdominal right lateral	33 (26.6)
Transabdominal left lateral	2 (1.6)
Transabdominal right dorsolateral	3 (2.4)
Transabdominal right dorsal	2 (1.6)
Transhepatic right lateral	22 (17.7)
Transhepatic ventral	17 (13.7)
Transhepatic right ventrolateral	3 (2.4)

^1^: median (25%-; 75%-quartiles) (range), ^2^: mean value ± standard deviation (range), ^3^: numbers (percentage).

**Table 3:** Complications occurring in the peri-interventional period as outlined by the SIR.

Type of Complication (Category)	Interventions (n, %)
***Minor complication:***	*5 (5.5%)*
Bloody aspirate (A)	1 (1.1%)
Small pneumothorax (A)	3 (3.3%)
Mild hemorrhage (A)	1 (1.1%)
***Major complication:***	*2 (2.2%)*
Mild hemorrhage (C)	1 (1.1%)
Severe hemorrhage (D)	1 (1.1%)

n: number; %: percentage. A: SIR category “No therapy, no consequence”. C: SIR category “Requires therapy, minor hospitalization (<48 h)”. D: SIR category “Requires major therapy, unplanned increase in level of care, prolonged hospitalization (>48 h)”.

**Table 4:** Details of the parameters calculated in the generalized linear mixed models depicted in Figure 6.

	CRP			Leukocyte Count			Interleukin-6		
*Predictors*	*Estimates*	*CI*	*p*	*Estimates*	*CI*	*p*	*Estimates*	*CI*	*p*
(Intercept)	0.62	0.47–0.77	**<0.001**	0.82	0.74–0.90	**<0.001**	1.99	1.97–2.23	**<0.001**
Time (days)	−0.01	−0.01–−0.01	**<0.001**	−0.00	−0.01–−0.00	**<0.001**	−0.01	−0.03–0.00	**0.019**
**Random Effects:**									
σ^2^	0.07			0.02			0.13		
τ_00_	0.13 _Procedure ID_			0.04 _Procedure ID_			0.15 _Procedure ID_		
ICC	0.64			0.69			0.54		
N	25 _Procedure ID_			25 _Procedure ID_			15 _Procedure ID_		
Observations	406			421			71		
Marginal R^2^/ Conditional R^2^	0.029/0.653			0.027/0.694			0.054/0.567		

CI: Confidence Interval; R^2^: Coefficient of Determination; σ^2^: distribution-specific variance; τ_00_: between-subject-variance; ICC: intraclass correlation coefficient, N: number of subjects. *p*-values in bold indicate significant effects. CRP: C-reactive protein.

**Table 5:** Success rates for fluid collections with and without positive microbial detection employing the criterion of decreasing inflammatory values.

	C-Reactive Protein			Leukocytes			Interleukin-6		
Fluid Collection Infection Status	Elevated (n)	Success (n, %)	No Success (n, %)	Elevated (n)	Success (n, %)	No Success (n, %)	Elevated (n)	Success (n, %)	No Success (n, %)
Infected	47	31 (65.9)	16 (34.1)	19	15 (78.9)	4 (21.1)	22	13 (59.1)	9 (40.9)
Non-infected	23	14 (60.9)	9 (39.1)	8	6 (75.0)	2 (25.0)	11	5 (45.5)	6 (54.5)
Total	70	45 (64.3)	25 (35.7)	27	21 (77.8)	6 (22.2)	33	18 (54.5)	15 (45.5)

n: number; %: percentage.

## Data Availability

The data presented in this study are available upon reasonable request from the corresponding author.

## Supplementary Materials

The following supporting information can be downloaded at: https://www.mdpi.com/article/10.3390/diagnostics14040353/s1, Table S1: Parameters of the generalized linear mixed models (GLMM) used in Supplementary Figure S1; Table S2: Parameters of the generalized linear mixed models (GLMM) used for time course of parameters for liver function; Table S3: Parameters of the generalized linear mixed models (GLMM) used for time course of parameters for liver damage; Table S4: Visual appearance of the drainage fluid depending on the infection status; Table S5: Factors affecting clinical success (Level of intervention sessions); Table S6: Factors affecting clinical success (Level of fluid collections); Table S7: Factors affecting clinical success (Level of inserted drainages); Figure S1: Development of parameters for liver function and liver damage within 30 days after the intervention in subjects with no evidence of further surgical interventions or complications in the patient record; Figure S2: Microbiological results.
