# Supplementary material for: Technical and Clinical Outcome of Low-Milliampere CT Fluoroscopy-Guided Percutaneous Drainage Placement in Abdominal Fluid Collections after Liver Transplantation: A 16-Year Retrospective Analysis of 50 Consecutive Patients
Source: Diagnostics (Basel). 2024 Feb 6;14(4):353. doi: 10.3390/diagnostics14040353 (PMC10887879; doi:10.3390/diagnostics14040353)
Supplement: Supplementary file 1 [file diagnostics-14-00353-s001.zip › supplementary_tables_v2_RS.pdf]

diagnostics

MDPI

Supplementary Table S1: Parameters of the generalized linear mixed models (GLMM) used in Supplementary Figure S1.

	Albumin			Cholinesterase			ALT		
Predictors	*Estimates*	*CI*	*p*	*Estimates*	*CI*	*p*	*Estimates*	*CI*	*p*
(Intercept)	0.44	0.39 – 0.49	**<0.001**	0.41	0.33 – 0.48	**<0.001**	1.44	1.28 – 1.60	**<0.001**
Time (days)	0.00	0.00 – 0.00	**<0.001**	0.01	0.00 – 0.01	**<0.001**	-0.01	-0.01 – -0.00	**<0.001**
**Random Effects:**									
σ^2	0.00			0.00			0.05		
τ_{00}	0.01 Procedure ID			0.03 Procedure ID			0.15 Procedure ID		
ICC	0.81			0.86			0.77		
N	19 Procedure ID			24 Procedure ID			25 Procedure ID		
Observations	93			167			364		
Marginal R^2 / Conditional R^2	0.051 / 0.823			0.055 / 0.870			0.017 / 0.776		

CI: Confidence Interval; R^2: Coefficient of Determination; σ^2: distribution-specific variance; τ00: between-subject-variance; ICC: intra-class correlation coefficient, N: number of subjects. P-values in bold indicate significant effects. ALT: Alanine-Aminotransferase.

Supplementary Table S2: Parameters of the generalized linear mixed models (GLMM) used for time course of parameters for liver function.

	Bilirubin			INR		
Predictors	*Estimates*	*CI*	*p*	*Estimates*	*CI*	*p*
(Intercept)	0.11	-0.03 – 0.24	0.118	1.15	1.11 – 1.20	**<0.001**
Time (days)	-0.00	-0.00 – 0.00	0.085	-0.00	-0.00 – -0.00	0.068
**Random Effects:**						
σ^2		0.03			0.01	
τ_{00}		0.11 Procedure ID			0.01 Procedure ID	
ICC		0.79			0.63	
N		25 Procedure ID			22 Procedure ID	
Observations		365			282	
Marginal R^2 / Conditional R^2		0.002 / 0.795			0.006 / 0.636	

CI: Confidence Interval; R2: Coefficient of Determination; σ2: distribution-specific variance; τ00: between-subject-variance; ICC: intraclass correlation coefficient, N: number of subjects. P-values in bold indicate significant effects. INR: International Normalized Ratio.

Supplementary Table S3: Parameters of the generalized linear mixed models (GLMM) used for time course of parameters for liver damage.

	GGT			AST			Antithrombin		
Predictors	*Estimates*	*CI*	*p*	*Estimates*	*CI*	*p*	*Estimates*	*CI*	*p*
(Intercept)	2.10	1.94 – 2.25	**<0.001**	1.46	1.38 – 1.54	**<0.001**	1.90	1.78 – 2.02	**<0.001**
Time (days)	0.00	0.00 – 0.01	0.091	-0.00	-0.00 – 0.00	0.158	-0.00	-0.01 – 0.01	0.962
**Random Effects:**									
σ^2	0.03			0.03			0.00		
τ_{00}	0.14 Procedure ID			0.03 Procedure ID			0.02 Procedure ID		
ICC	0.82			0.57			0.81		
N	25 Procedure ID			24 Procedure ID			7 Procedure ID		
Observations	358			278			21		
Marginal R^2 / Conditional R^2	0.005 / 0.820			0.004 / 0.567			0.000 / 0.809		

CI: Confidence Interval; R2: Coefficient of Determination; σ2: distribution-specific variance; τ00: between-subject-variance; ICC: intraclass correlation coefficient, N: number of subjects. P-values in bold indicate significant effects. GGT: Gamma glutamyltransferase; AST: Aspartate Aminotransferase

Supplementary Table S4: Visual appearance of the drainage fluid depending on the infection status.

Visual appearance [1]		Proof of germs [2]	
		Positive	*Negative*
Bloody	8 (12.3%)	4 (50.0%)	4 (50.0%)
Chylus	**1 (1.5%)**	1 (100.0%)	0 (0.0%)
Purulent	**15 (23.1%)**	**14 (93.3%)**	1 (6.7 %)
Bilious	24 (36.9%)	13 (54.2 %)	11 (45.8%)
Serous	17 (26.2%)	9 (52.9 %)	8 (47.1 %)

[1]: Numbers (Percentage). The group includes all cases where the visual appearance of the drainage fluid was documented (n=65). Percentages refer to the column values. [2]: Numbers (Percentage). Subgroup in which germ detection was performed. Percentages refer to the row values. Values in bold indicates significant result in Chi^2-test.

Supplementary Table S5: Factors affecting clinical success. Parameters refer to the level of each intervention session.

**Parameter**	**Value / Unit**	**Clinical Success Group**				**P-value (Intergroup comparison)**	**P-value (Pairwise post-hoc comparison)** [1)]
		Sucessful	n	Unsuccessful	n		
Age							
	Years	50.6 ± 10.43 (29-67) [2)]	53	48.3 ± 11.27 (26-41) [2)]	13	0.645	
Gender							
	Males	42 (75.0%) [3)]	56	11 (84.6%) [3)]	13	0.717	
	Females	14 (25.0%) [3)]		2 (15.4%) [3)]			
Indication for liver transplantation							
	Ethyltoxic	18 (32.1%) [3)]	56	1 (7.7%) [3)]	13	**0.016**	**<0.0001**
	Ethyltoxic + HCC	6 (10.7%) [3)]		0 (0.0%) [3)]			**0.009**
	Other [*)]	32 (57.2%) [3)]		12 (92.3%) [3)]			>0.999
Transplantation number							
	First transplantation	50 (89.3%) [3)]	56	13 (100%) [3)]	13	0.586	
	Retransplantation	6 (10.7%) [3)]		0 (0.0%) [3)]			
Backtable vascular anastomosis required during transplantation surgery							
	Accessory / aberant hepatic artery	13 (28.9%) [3)]	45	1 (12.5%) [3)]	8	>0.999	
	none	32 (71.1%) [3)]		7 (87.5%) [3)]			
Type of Biliary anastomosis							
	End-to-end	31 (75.6%) [3)]	41	6 (75.0%) [3)]	8	0.815	
	End-to-side	3 (7.3%) [3)]		0 (0.0%) [3)]			
	Biliodigestive	7 (17.1%) [3)]		2 (25.0%) [3)]			
Type of donor liver							

	Whole liver	43 (84.3%) [3)]	51	10 (100.0%) [3)]	10	0.332
	Split liver	8 (15.7%) [3)]		0 (0.0%) [3)]		
Type of transplanation surgery						
	Piggyback	27 (64.3%) [3)]	42	5 (50.0%) [3)]	10	0.532
	Belghiti	11 (26.2%) [3)]		3 (30.0%) [3)]		
	Vena cava resection	4 (9.5%) [3)]		2 (20.0%) [3)]		
Additonal surgery procedures during transplantation						
	Performed	3 (5.9%) [3)]	51	2 (20.0%) [3)]	10	0.189
	Not performed	48 (94.1%) [3)]		8 (80.0%) [3)]		
Intervall Transplantation surgery to Intervention						
	Days	108.5 [34, 327] (7-8351) [4)]	56	162 [43, 687] (19-8315) [4)]	13	0.519
Applied Immunosupressiva						
	Tacrolimus	35 (62.5%) [3)]	56	8 (61.5%) [3)]	13	0.768
	Tacrolimus+Mycophenolate	5 (8.9%) [3)]		1 (7.7%) [3)]		
	Cyclosporin A	7 (12.5%) [3)]		3 (23.1%) [3)]		
	Cyclosporin A+ Mycophenolate	3 (5.4%) [3)]		0 (0.0%) [3)]		
	Cyclosporin A+ Sirolimus	2 (3.6%) [3)]		0 (0.0%) [3)]		
	Sirolimus	4 (7.2%) [3)]		1 (7.7%) [3)]		
Total number of fluid collections						
	1	38 (66.1%) [3)]	56	10 (76.9%) [3)]	13	
	2	16 (28.6%) [3)]		2 (15.4%) [3)]		
	3	2 (3.6%) [3)]		1 (7.7%) [3)]		
	4	1 (1.8%) [3)]		0 (0.0%) [3)]		

N: number; [1]: only if applicable; [2]: Mean value ± standard deviation (range), [3]: Numbers (Percentage); [4]: median [25%-; 75%-quartiles] (range), HCC: Hepatocellular carcinoma
*) For the sake of clarity, the remaining indications are not listed here. In these cases, no significant group differences were observed
p-Values in bold indicate significant differences

Supplementary Table S6: Factors affecting clinical success. Parameters refer to the level of the fluid collection.

Parameter	Value / Unit	Clinical Success Group				P-value	P-value
		Sucessful	n	Unsuccessful	n	(Intergroup comparison)	(Pairwise post-hoc comparison) 1)
Contrast enhancement on prior CT	Enhancement	12 (25.0%) 2)	48	6 (50.0%) 2)	12	0.156	
	No Enhancement	36 (75.0%) 2)		6 (50.0%) 2)			
Air entrapment on prior CT	Entrapment	16 (23.2%) 2)	69	3 (18.8%) 2)	16	>0.999	
	No Entrapment	53 (76.8%) 2)		13 (81.2%) 2)			
Max. diameter of fluid collection		7.25 [4.2, 10.6] (2-17.2) 3)	72	6.3 [5.25, 11.2] (2.7-14.1) 3)	16	0.871	
Location of fluid collection	Intrahepatic right liver lobe	32 (44.4%) 2)	72	7 (43.8%) 2)	16	0.527	
	Intrahepatic left liver lobe	11 (15.3%) 2)		2 (12.5%) 2)			
	Intrahepatic central	3 (4.2%) 2)		0 (0.0%) 2)			
	Centrally congested bile ducts	2 (2.8%) 2)		0 (0.0%) 2)			
	Prehepatic	5 (6.9%) 2)		1 (6.3%) 2)			
	Subhepatic	8 (11.1%) 2)		3 (18.8%) 2)			
	Perihepatic medial	5 (6.9%) 2)		0 (0.0%) 2)			
	Perihepatic lateral	4 (5.6%) 2)		1 (6.3%) 2)			
	Retrohepatic right	0 (0.0%) 2)		2 (12.5%) 2)			
	Retrohepatic left	1 (1.4%) 2)		0 (0.0%) 2)			
	Subphrenic	1 (1.4%) 2)		0 (0.0%) 2)			
Access to collection	Direct	28 (40.0%) 2)	70	4 (30.8%) 2)	13	0.758	
	Transhepatic	42 (60.0%) 2)		9 (69.2%) 2)			
Visual appearance of aspirate	Bilious	21 (36.2%) 2)	58	3 (23.1%) 2)	13	**0.018**	>0.999
	Bloody	4 (6.9%) 2)	58	6 (46.2%) 2)	13		**0.002**
	Chylus	3 (5.2%) 2)	58	0 (0.0%) 2)	13		>0.999
	Purulent	15 (25.9%) 2)	58	2 (15.4%) 2)	13		>0.999
	Serous	15 (25.9%) 2)	58	2 (15.4%) 2)	13		>0.999

Type of fluid collection							
	Abscess	27 (43.5%)	62	9 (75.0%)	12	**0.040**	**<0.0001**
	Biloma	23 (37.1%)		1 (8.3%)			**0.002**
	Haematoma	4 (6.5%)		2 (16.7%)			**0.008**
	Seroma	8 (12.9%)		0 (0.0%)			0.052
Volume of aspirate							
	ml	100 [10,300] (3-600) [3)]	13	20 [11.5, 510] (3-1000) [3)]	3	0.946	

N: number; [1]: only if applicable; [2]: Numbers (Percentage); 3: median [25%-; 75%-quartiles] (range)

p-Values in bold indicate significant differences

Supplementary Table S7: Factors affecting clinical success. Parameters refer to the level of the inserted drainages.

Parameter	Value / Unit	Clinical Success Group				P-value (Intergroup comparison)	P-value (Pairwise post-hoc comparison) [1)]
		Sucessful	n	Unsuccessful	n		
Total number of drains							
	1	69 (89.6%) [2)]	77	15 (88.2%) [2)]	17	>0.999	
	2	8 (10.4%) [2)]		2 (11.8%) [2)]			
Access path							
	Transabdominal ventral	19 (24.7%) [2)]		4 (23.5%) [2)]			>0.999
	Transabdominal right ventrolateral	7 (9.1%) [2)]		3 (17.6%) [2)]			>0.999
	Transabdominal right lateral	18 (23.4%) [2)]		0 (0.0%) [2)]			**0.042**
	Transabdominal left lateral	2 (2.6%) [2)]		0 (0.0%) [2)]			>0.999
	Transabdominal right dorsolateral	3 (3.9%) [2)]	77	0 (0.0%) [2)]	17	**0.031**	>0.999
	Transabdominal right dorsal	0 (0.0%) [2)]		2 (11.8%) [2)]			>0.999
	Transhepatic right lateral	15 (19.5%) [2)]		4 (23.5%) [2)]			>0.999
	Transhepatic ventral	10 (13.0%) [2)]		4 (23.5%) [2)]			>0.999
	Transhepatic right ventrolateral	3 (3.9%) [2)]		0 (0.0%) [2)]			>0.999
Technique							
	Direct	73 (96.1%) [2)]	76	16 (100.0%) [2)]	16	>0.999	
	Seldinger	3 (3.9%) [2)]		0 (0.0%) [2)]			
Drain size							
	7.5F	9 (11.7%) [2)]		0 (0.0%) [2)]			
	8F	23 (29.9%) [2)]		5 (29.4%) [2)]			
	10F	36 (46.8%) [2)]	77	10 (58.8%) [2)]	17	0.799	
	12F	8 (10.4%) [2)]		2 (11.8%) [2)]			
	14F	1 (1.3%) [2)]		0 (0.0%) [2)]			
Drain duration							
	Days	7 [6, 19] (2-54) [3)]	29	10 [10,15] (8-54) [3)]	5	0.251	

N: number; [1]: only if applicable; [2]: Numbers (Percentage); 3: median [25%-; 75%-quartiles] (range)

p-Values in bold indicate significant differences
