# Supplementary figures and images for: Technical and Clinical Outcome of Low-Milliampere CT Fluoroscopy-Guided Percutaneous Drainage Placement in Abdominal Fluid Collections after Liver Transplantation: A 16-Year Retrospective Analysis of 50 Consecutive Patients
Source: Diagnostics (Basel). 2024 Feb 6;14(4):353. doi: 10.3390/diagnostics14040353 (PMC10887879; doi:10.3390/diagnostics14040353)

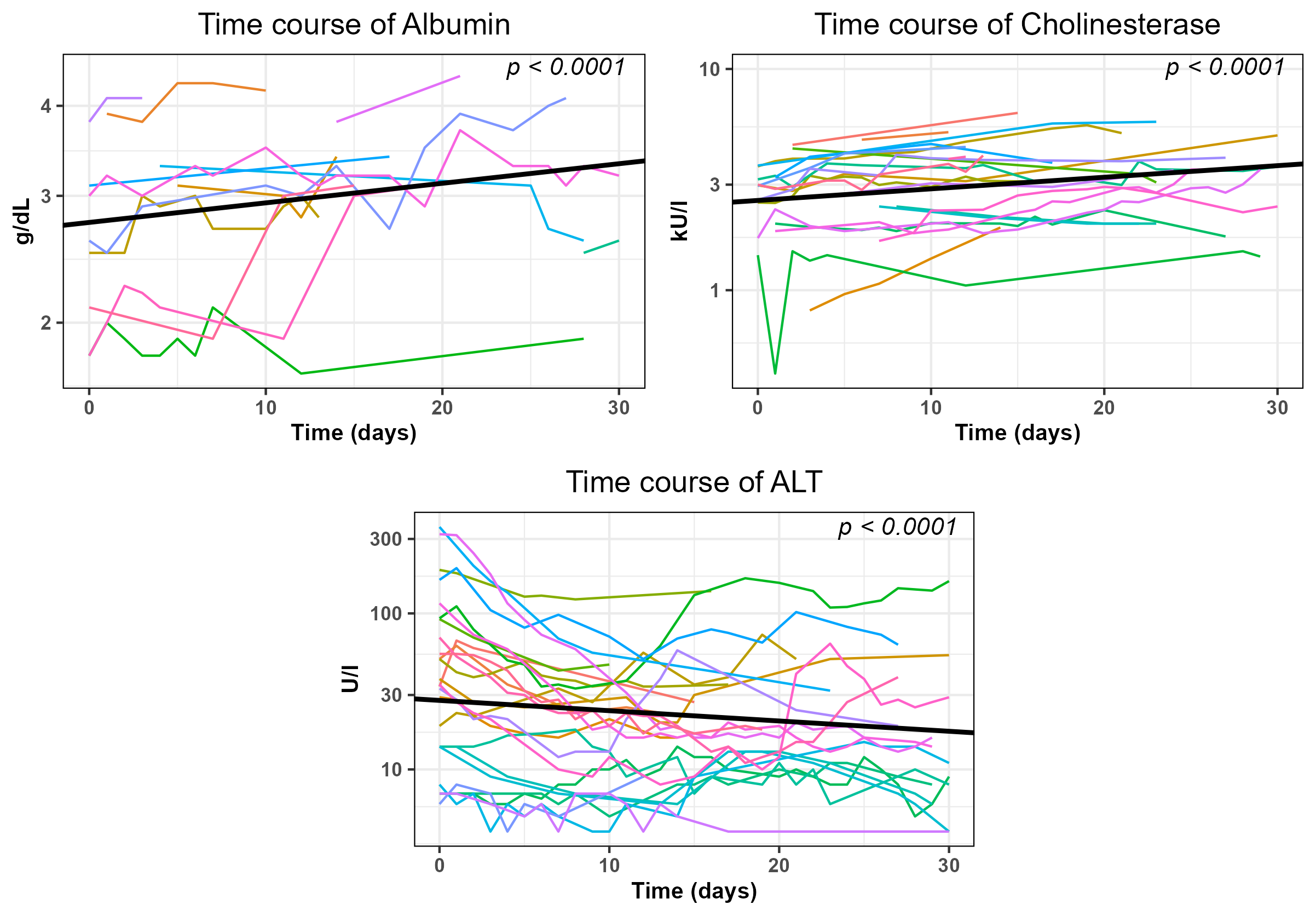

Supplement: Supplementary file 1 [file diagnostics-14-00353-s001.zip › supplementary_figure_S1.tiff]

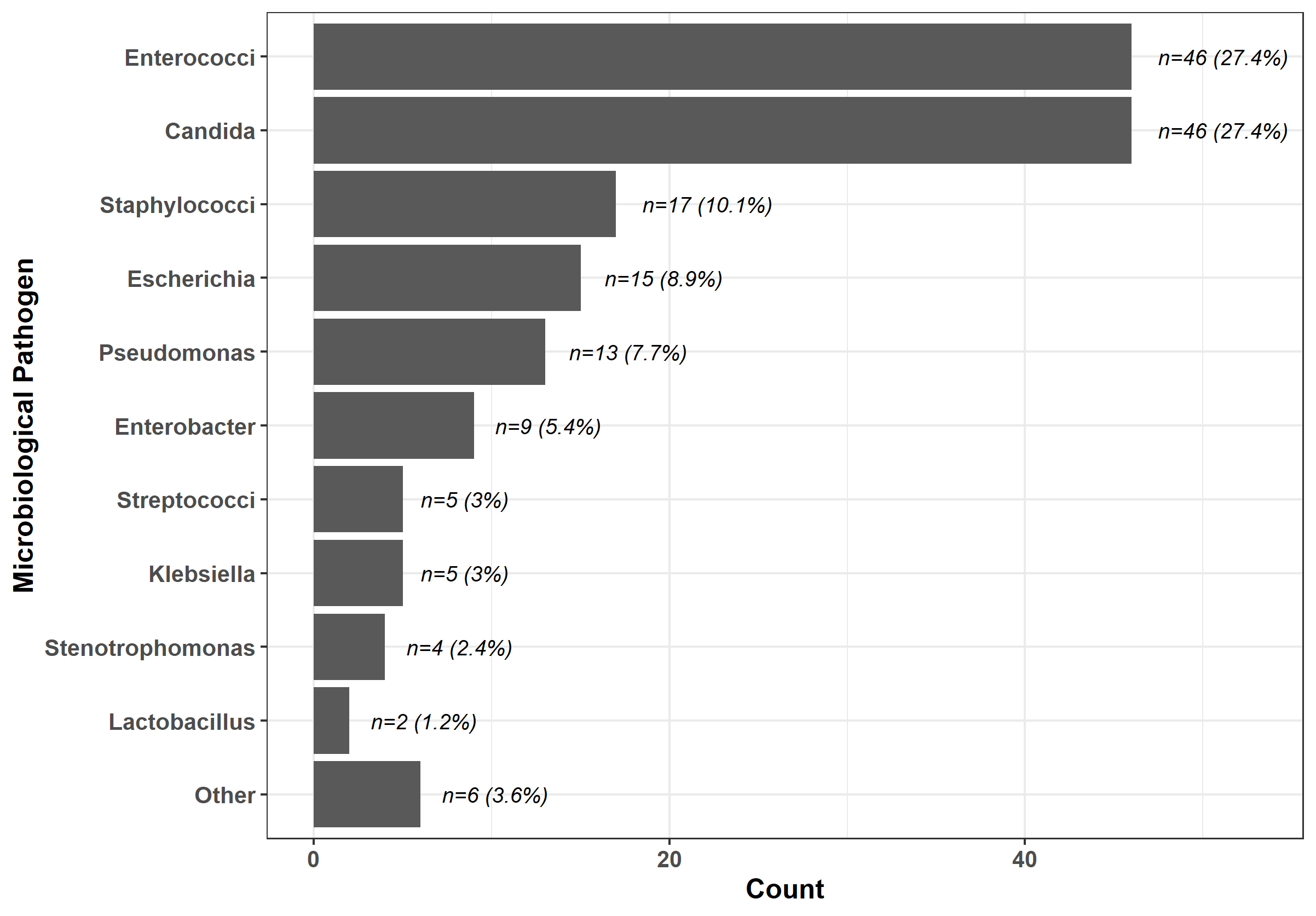

Supplement: Supplementary file 1 [file diagnostics-14-00353-s001.zip › supplementary_figure_S2.tiff]
